# Exploring the cortical involvement in sensorimotor integration during early stages of independent walking

**DOI:** 10.1007/s00221-025-07099-4

**Published:** 2025-05-26

**Authors:** Ruud A. J. Koster, Coen S. Zandvoort, Jennifer N. Kerkman, Andreas Daffertshofer, Nadia Dominici

**Affiliations:** 1 Department of Human Movement Sciences, Faculty of Behavioural and Movement Sciences, Vrije Universiteit Amsterdam, Van der Boechorststraat 9, Amsterdam, 1081BT The Netherlands; 2 Institute for Brain and Behaviour Amsterdam, Vrije Universiteit Amsterdam, Van der Boechorststraat 9, Amsterdam, 1081BT The Netherlands; 3 Amsterdam Movement Sciences, Rehabilitation & Development, Amsterdam, The Netherlands; 4 Department of Paediatrics, University of Oxford, Oxford, UK

**Keywords:** Gait development, Muscle synergies, Cortico-muscular coherence, Toddlers, Motor control

## Abstract

The control of human locomotion is governed by a combination of congenital and emerging locomotor muscle synergies. The first arguably build on spinal and brainstem circuitries, whereas the latter have a cortical resemblance. By hypothesis this cortical activity reflects sensorimotor integration which matures during the development of walking. We therefore investigated the role of sensory information by manipulating the gravitational loading in 23 toddlers walking on an instrumented treadmill while recording 3D kinematics, EEG, and EMG of 24 trunk and lower extremity muscles. Sensory loading was manipulated via low and high levels of external body weight support. Cortico-synergy connectivity was compared between the two different support levels and at two stages of gait development: onset of independent walking (just after the first steps) and at six months of walking experience. These two age groups consisted of different subjects. For twelve toddlers data quantity and quality met requirements to enter analysis. Four muscle synergies sufficed to characterise gait, regardless of support level and developmental stage. Cortico-synergy coherence confirmed involvement of the sensorimotor cortex only in the two emerging synergies associated with walking onset. Reduced sensory loading was accompanied by a decreased coherence but only in toddlers with little walking experience. That gravitational loading alters the cortical resemblance of the synergies, especially at an early age, suggests that it reflects the integration of sensory information, at least to some extent. Our findings hint at the importance of sensorimotor integration in the emergence of the synergies linked to the onset of independent walking.

**Supplementary Information:**

The online version contains supplementary material available at 10.1007/s00221-025-07099-4.

## Introduction

Human locomotion demands intricate motor control. It typically takes more than a year of development to reach the stage where toddlers can walk independently (Sugden et al. 2013). Already in new-borns a coordinated alternating stepping response can be observed (Dominici et al. 2011; Forssberg 1985; McGraw 1940; Yang et al. 1998). This involuntary response only manifests when a certain level of loading is applied to the legs (Thelen 1986; Thelen and Fisher 1982). Even at this rudimentary stage of gait development, loading-related information seems vital in facilitating motor control. Gravitational loading provides valuable sensory information through different modalities: foot sole pressure activates cutaneous mechanoreceptors, stress in and activation of anti-gravity muscles activate muscle spindles, and accompanying tendon tension activates Golgi tendon organ feedback. This sensory information is involved in motor control in both newborns and adults.

During development gait control continues to improve the integration of different sensory pathways (visual, vestibular, proprioceptive, and tactile) (Lam et al. 2003; Lamb and Yang 2000; Pang and Yang 2000, 2001; Thelen et al. 1987; Yang et al. 1998, 2005). When toddlers take their first independent steps, reduced gravitational loading has been shown to influence the behaviour and control of gait (Dominici et al. 2007; Kerkman et al. 2022). This is accompanied by an overshoot in foot swing when body weight is partially supported, indicating the yet limited ability to integrate loading changes (Dominici et al. 2007). When gait is fully developed, however, different levels of loading barely affect kinematic parameters and are only detectable in kinetics and muscle activity (Apte et al. 2018; Dominici et al. 2007; Finch et al. 1991; Ivanenko et al. 2002, 2004). Hence it seems safe to say that motor control in developing and developed gait is distinctively affected by reduced gravitational loading information.

Gait typically involves a set of only four muscle synergies (also referred to as locomotor primitives or motor modules) in the trunk and lower limbs (Dominici et al. 2011). A synergy is composed of a coordinated activity of multiple muscles. A distinct set of weights indicates the contribution of the individual muscles to the synergy which shows a characteristic temporal activation present in all contributing muscles. The neuroanatomical basis of such motor modules has been studied extensively in both humans and other animals (D’Avella et al. 2003; Dominici et al. 2011; Giszter et al. 1998; Han et al. 2013; Jin et al. 2023; Kargo and Giszter 2008; Kutch and Valero-Cuevas 2012; Mussa-Ivaldi et al. 1994; Smith et al. 2017). Findings point to central pattern generator structures (CPGs) in the spinal cord (Hart and Giszter 2010; Lemay and Grill 2004; Pinto and Golubitsky 2006; Saltiel et al. 2001; Sun et al. 2022; Yang et al. 2019) but cortical involvement is essential for these synergies, particularly in bipedal human gait (Capaday 2002; Dietz 2002; Nielsen 2003; Zandvoort et al. 2022). More recently, the development of synergies has been a focus point for studies. There is evidence of synergies being present at birth (Dominici et al. 2011; Yang et al. 2019), but new synergies can also form during development (Dominici et al. 2011; Sylos-Labini et al. 2020).

For human locomotion, two out of the four muscle synergies can be considered congenital. They are active during the single support phase and contribute to the stepping response observed in new-borns (Dominici et al. 2011; Sylos-Labini et al. 2020; Thelen and Cooke 1987). Presumably they rely on sub-cortical structures, particularly spinal circuitries, but also on deeper cerebellar and brainstem networks (Dominici et al. 2011; Zandvoort et al. 2021). In contrast, the other two synergies, which are active during the double support phase, emerge as toddlers begin to walk independently. While the development of these emerging synergies is still poorly understood, the onset of independent walking is known to require a certain level of cortical maturation (Garwicz et al. 2009; Grillner 2011). This suggests that cortical involvement plays a role in the onset of gait and, by extension, the emerging synergies. Unlike the congenital synergies, the emerging ones exhibit significant coherence with cortical activity, particularly in the sensorimotor, supplementary motor, and premotor areas shortly after foot contact (Zandvoort et al. 2021, 2022). We here speculate that the coherence reflects a cortical drive that resembles the integration of sensory information and facilitates the guidance of motor output.

Put differently, sensorimotor integration may play a pivotal role in shaping emerging locomotor synergies by enabling the cortex to adaptively coordinate and refine motor patterns based on sensory feedback. This is in line with findings of the spinal cord as the locomotor synergies’ neural substrate with reliance on a cortical contribution. Cheung et al. (2024) proposed a model for the development of muscle synergies. It builds on an initial cortical drive to spinal interneurons (the CPGs) and individual muscles, as well as on afferents from the muscles back to the interneurons. It assumes muscles are initially voluntarily coactivated via cortical sensorimotor processing. That is, also in this model cortical sensorimotor processing seemingly forms the basis for the development of these emerging synergies. The presence of a cortical resemblance of the emerging synergies in adults (Zandvoort et al. 2022), whose synergies are already robustly formed (Cappellini et al. 2020; Chvatal and Ting 2013; Torres-Oviedo and Ting 2010), may suggest that the role of the cortical contribution adapts during development.

With the current experiment, we explored the role of cortical sensorimotor processing in locomotor synergies and their development. We altered loading-related sensory information through gravitational unloading in toddlers at two stages of gait development: immediately following the onset of independent walking and after 6 months of walking experience. We hypothesised that congenital synergies would function without cortical contribution. In contrast, we expected emerging locomotor synergies to show connectivity with the sensorimotor cortex, and that this connectivity peaks during the sensory information-laden double support phase. A reduced availability of sensory information was expected to lessen the cortical contribution, particularly in the group with the least independent walking experience where it may align with a failure to properly adapt gait control (Dominici et al. 2007).

## Methods

### Subjects

Twenty-three typically developing toddlers were recruited through word-of-mouth. Some of them had been included in our previous study (Kerkman et al. 2022). Subjects with known neurological and developmental diseases were excluded. Recordings were conducted at two stages of development: (1) as inexperienced walkers, right after ‘walking onset’, i.e., when the toddler had just started taking their first steps independently (referred to as FS for First Steps); and (2) as experienced walkers, at an ‘independent walking age’ of six months (referred to as the FS + 6 group). To capture the first independent steps, regular communication was established with the parents to monitor their toddler’s walking ability. Recording sessions of the first independent steps were scheduled when parents reported that their toddler was able to walk independently for at least four consecutive steps.

The procedures adhered to the Declaration of Helsinki. The experimental protocol had been approved by the local ethical committee of the Faculty of Behavioural and Movement Sciences Amsterdam (#VCWE-2016-082 and #VCWE-2017-108) and the toddlers’ legal guardians provided written informed consent prior to participation. The measurements were conducted at the BabyGaitLab in the Department of Human Movement Sciences at the Vrije Universiteit Amsterdam, with at least one legal guardian present. Our laboratory settings and procedures were designed to ensure that potential risks were no greater than those encountered during regular walking at home.

### Experimental setup

Upon entering the laboratory, the toddlers were given time to acclimate themselves to the laboratory settings and researchers before the start of the experiment. The experimental procedure involved comfortable walking, with toddlers being encouraged to take steps while being supported by their hands or trunk over a moving instrumented treadmill. Treadmill speed was tuned individually for each toddler to elicit continuous stepping movements resembling their natural overground walking pattern, judged by the researchers and parents. Alongside the walking trials, an additional trial was conducted during each session wherein the toddler remained standing or sat quietly on the non-moving instrumented treadmill for at least two seconds to measure their body weight (weighting trial).

Targeting effects of load bearing, the toddlers were encouraged to walk on a treadmill while experiencing various levels of external body weight support (BWS). Manual unloading is a standard procedure used extensively in infants (Dominici et al. 2007; Kerkman et al. 2022; Lam et al. 2003; Pang and Yang 2000; Vasudevan et al. 2016). In brief, the experimenter was seated on a bench behind the toddler and provided a firm support to the toddler’s trunk using both hands, maintaining a relatively constant vertical force during several consecutive strides (see *Supplementary Material SM1)*. The treadmill was set at a comfortable speed per subject (FS: 0.8 ± 0.2 km/h; FS + 6: 1.1 ± 0.3 km/h). BWS was varied trial by trial to cover a wide range of support levels for every participant. We recorded as many strides as feasible at different BWS-levels.

### Data acquisition

Kinematic and video data were recorded with a Vicon motion capture system sampled at 100 Hz (ten Vicon vero v2.2 cameras placed around the treadmill and one Vue Vicon camera placed in the sagittal plane, Oxford, UK). Kinematics were captured by attaching twenty reflective markers (∅ 14 mm) to the toddler’s body with adhesive tape (Bekius et al. 2021a). Here, we focused on the markers attached to the lateral malleoli and fifth metatarsal-phalangeal joints for gait events. Vertical ground reaction forces were recorded at 1000 Hz with a resolution of 3 N using a treadmill mounted force plate (N-Mill 60 × 150 cm, Motek Medical BV, Amsterdam, the Netherlands).

Muscular activity was collected bilaterally from twelve muscles of the legs and back: tibialis anterior (TA), gastrocnemius medialis (GM), gastrocnemius lateralis (GL), soleus (SOL), rectus femoris (RF), vastus medialis (VM), vastus lateralis (VL), biceps femoris (BF), semitendinosus (SEM), tensor fasciae latae (TFL), gluteus maximus (GLM), and erector spinae at L2 level (ES). We used two Cometa Mini Wave Wireless 16-channels EMG systems (Cometa srl, Italy) with online band-pass filtering between 10 and 500 Hz and sampling at 2000 Hz. Reusable mini golden surface EMG disc–electrode pairs (15-mm-diameter electrodes, acquisition area of 4 mm^2^) were placed at the approximate location of the muscle belly according to standard recommendations for minimizing crosstalk between adjacent muscles (Hermens et al. 2000). To ensure data quality and minimize movement artifacts, the skin was cleansed with alcohol prior to electrode placement. The EMG sensor units were attached to the skin using tape and secured with body elastic gauze (Bekius et al. 2021a; Zandvoort et al. 2022).

EEG was recorded using 32-electrode EEG caps in the standard 10–20 montage (WaveGuard^™^, ANT-Neuro, Enschede, the Netherlands). Impedance gel (SonoGel, Bad Camberg, Germany) was applied to the EEG cap to minimise the impedance between the electrodes and the skin. The gel was applied to the cap before it was mounted on the head to reduce discomfort (Zandvoort et al. 2022). After cap placement and alignment to the fiducials, the impedance was improved by redistributing the gel with a small blunt probe. If necessary, additional gel was injected to keep the impedance below 50 kW. The cortical activity was recorded with the CPz electrode as reference and sampled at 2048 Hz (eego^™^ mylab, ANT B.V., Enschede, the Netherlands). Upon completion of the preparation, toddlers were given some time to settle before the experimental procedure started.

### Data analysis

Data processing was performed using Matlab (2021b The MathWorks, Natick, MA, USA) and the FieldTrip toolbox (Oostenveld et al. 2011). We included full strides, defined as right foot contact to subsequent right foot contact. All strides were marked by identifying foot contact and foot off events for both legs through visual inspection using the digital video recordings and the marker trajectories from Vicon Nexus software (Vicon, Oxford, UK). An experienced researcher assessed the gait events while monitoring the kinematic data of the foot markers. Specifically, foot contact events were confirmed by identifying (local) minima of the vertical displacement of either the lateral malleoli or the fifth metatarsophalangeal joint marker, depending on the initial point of foot-ground contact. Foot off events were validated by observing an increase in the vertical displacement of the lateral malleoli or the fifth metatarsophalangeal joint marker after it reached its lowest point during stance phase. This methodology aligns with previous studies (Cappellini et al. 2016; Dominici et al. 2007; Ivanenko et al. 2005, 2007; La Scaleia et al. 2018). We considered a sequence of strides successful if at least three consecutive strides were present. Strides when gait was initiated or halted, as well as strides deemed bad by visual inspection (running, jumping, interruptions, other behaviour), were excluded from further analysis.

#### Body weight support

The mean vertical force was determined per gait cycle. For each stride, the amount of (external) BWS was estimated as the reduction of mean vertical force over a gait cycle relative to body weight (as measured during the weighting trial; see *Supplementary Material SM1*). Using the BWS-levels per stride, we grouped strides in low and high BWS conditions (< 30% and > 55% of body weight, respectively). We chose these thresholds because support below 30% of body weight has been reported to have limited effect on kinematic and spatiotemporal gait parameters (Apte et al. 2018), while support exceeding 40% of body weight significantly alters foot coordination (Dominici et al. 2007) and muscle networks (Kerkman et al. 2022) in toddlers. We increased the latter to > 55% to ensure a clear distinction between conditions.

#### Pre-processing EEG & EMG

Bad EEG channels (defined as any channel in a recording exceeding ± 500 mV or with a standard deviation (SD) > 20× the median SD) were replaced by a distance weighted average of its neighbouring channels. Spikes in the data (defined as ± 2 ms around values exceeding ± 50 mV or more than 10 SDs from the mean, after subtracting either a 3s median-filtered or 10 Hz high-pass IIR-filtered version of the signal from itself) were removed through linear interpolation. Subsequently, the EEG was re-referenced to average reference. Then, we bandpass filtered the signals (1–200 Hz, bidirectional 2nd order Butterworth) and applied notch filters to remove line noise (50 Hz and its harmonics up to the Nyquist frequency, ± 0.5 Hz bandwidth, bidirectional 3rd order Butterworth). Additionally, independent component analysis served for artefact removal (Snyder et al. 2015). An independent component was considered an artefact when its temporal component’s median frequency was lower than 2 Hz or exceeded 80 Hz as that indicates movement artefacts(Kline et al. 2015) or muscle activity (Farella et al. 2002; Kumar et al. 2001), respectively, or if its spatial components were dominated by prefrontal or occipital channels, seminal for eye movements or neck muscle activity, respectively (Jung et al. 1998). Finally, the EEG was mean centred over time.

The EMG was pre-processed using the same band-pass and notch filters as the EEG. Subsequently, we estimated the signals’ Hilbert amplitude (Boonstra and Breakspear 2012) to obtain the broadband rectified EMG (EMG_r_). We also obtained EMG envelopes (EMG_e_) by subsequently applying a 30 Hz high-pass filter (bidirectional 2nd order Butterworth), Hilbert rectification, and 5 Hz low-pass filter (bidirectional 2nd order Butterworth filter).

#### Gait cycle time normalisation

Strides with low and high BWS (respectively, < 30% and > 55% of body weight support) were extracted and normalised in time from 0 to 100% of the gait cycle (defined as right foot contact to the consecutive right foot contact). We time-warped the data using linear interpolation such that the foot contact and foot off events of each leg and stride coincided with the average occurrence of that event in all strides. By doing so, the relative duration of the sub-phases (i.e., 1st double support, single stance, 2nd double support, and swing) was the same in every stride and heterogeneity within the gait cycle was corrected for. The normalised time axis contained 201 samples.

#### Muscle synergy analysis

Per subject, the time normalised EMG_e_ envelope of each muscle was amplitude normalised to its mean value over the low BWS strides, after which the subject average envelope per muscle was computed separately for low and high BWS. Then, per BWS condition we determined the average envelopes for the FS and FS + 6 groups and applied a non-negative matrix factorization (NNMF) to estimate the muscle synergy activation patterns and their corresponding weighting coefficients per BWS condition and group. We factorised the averaged EMGs, rather than averaging the factorised EMGs, to prevent potential biases in the synergies that may result from missing data. NNMF decomposes the input EMG matrix into a (small) number of temporal activation patterns and the corresponding weighting coefficients using:

1 $$\:{{EMG}_{e}}_{[T\times\:M]}={Y}_{[T\times\:S]}{W}_{[S\times\:M]}+\epsilon$$

where $\:Y$ comprises the temporal activation patterns; $\:W$ the corresponding weighting coefficients; $\:\epsilon$ the residual error; and $\:M$, $\:S$, and $\:T$ denote the numbers of muscles (= 24), synergies (= 4), and time points (= 201), respectively. The number of synergies was set to $\:S=4$ in accordance with the literature on toddler gait where the same numbers of muscles were reported (Bekius et al. 2021b; Dominici et al. 2011; Kerkman et al. 2022; Zandvoort et al. 2022). We employed an alternating least square iterative algorithm that we terminated whenever the change in size of either the residual or elements $\:Y$ and $\:W$ dropped below 10^− 4^, or upon reaching 100 iterations. The optimisation was repeated 10 times with random initial conditions to avoid spurious results. NNMF was applied to EMG envelopes averaged over strides and subjects because the number of strides per subject and condition varied substantially (Table 1). Hence, applying the NNMF over a concatenation of the strides would be biased towards the subject and conditions with the most strides. To quantify the quality of this analysis we defined the reconstruction accuracy (RA) of synergies via the fraction of the data’s Frobenius norm accounted for by the synergies:

2 $$\:RA=1-\frac{{\left\| {{EMG}_{e}-YW} \right\|}_{F}}{{\left\| {{EMG}_{e}} \right\|}_{F}}$$

The NNMF was performed per age group and BWS condition, though this hardly differed from the analysis of the averaged data pooled over all conditions (see *Supplementary Material SM4)*.

Finally, the weighting coefficients $\:{W}_{[S\times\:M]}$ quantify the contributions of synergy $\:s\:$to EMG signal $\:m$. We estimated how strong EMG $\:m$ contributed to synergy $\:s$ using the Penrose pseudo-inverse of $\:W$ and multiplied it with the (not time normalised) broadband EMG_r_ to reconstruct the (virtual) broadband temporal patterns of the synergies via:

3 $$\:{X}_{[t\times\:S]}={{EMG}_{r}}_{[t\times\:M]}{W}_{\left[M\times\:S\right]}^{-1}$$

These (virtual) activation patterns $\:{X}_{[t\times\:S]}$ entered the coherence analysis with the EEG (Zandvoort et al. 2019, 2021, 2022). The weighting coefficients obtained from EMG_e_ were used because the lowpass filtered signal gives a more reliable estimation of how active each muscle is at a given time, while the virtual temporal activation obtained by using the EMG_r_ ensured it has the necessary broadband frequency range to identify coherence.

#### Spectral estimates

The gait cycle consists of multiple phases during which synergies are distinctively active. To identify whether a synergy exhibited activity coherent with cortical signals, we first pooled the data of all conditions. For each stride, we estimated the power- and cross-spectra of, and between, (not time normalised) EEG and the muscle synergies’ (virtual) activation patterns as a function of time and frequency using Morlet wavelets with a width of seven periods. The resulting time-frequency spectra were then normalised to represent 0 to 100% of the gait cycle – see above. Finally, we determined the coherence matrices for each synergy according to:

4 $$\:{C}_{xy}(t,f)=\frac{{\left|{S}_{xy}(t,f)\right|}^{2}}{{S}_{xx}\left(t,f\right)\:{S}_{yy}(t,f)}$$

where $\:{C}_{xy}$ denotes the magnitude squared coherence, $\:{S}_{xy}$ the cross-spectral density between the 32 EEG channels $\:x$ and the activation pattern of synergy $\:y$, and $\:{S}_{xx}$ & $\:{S}_{yy}$ representing the corresponding power spectral densities.

**Fig. 1:**
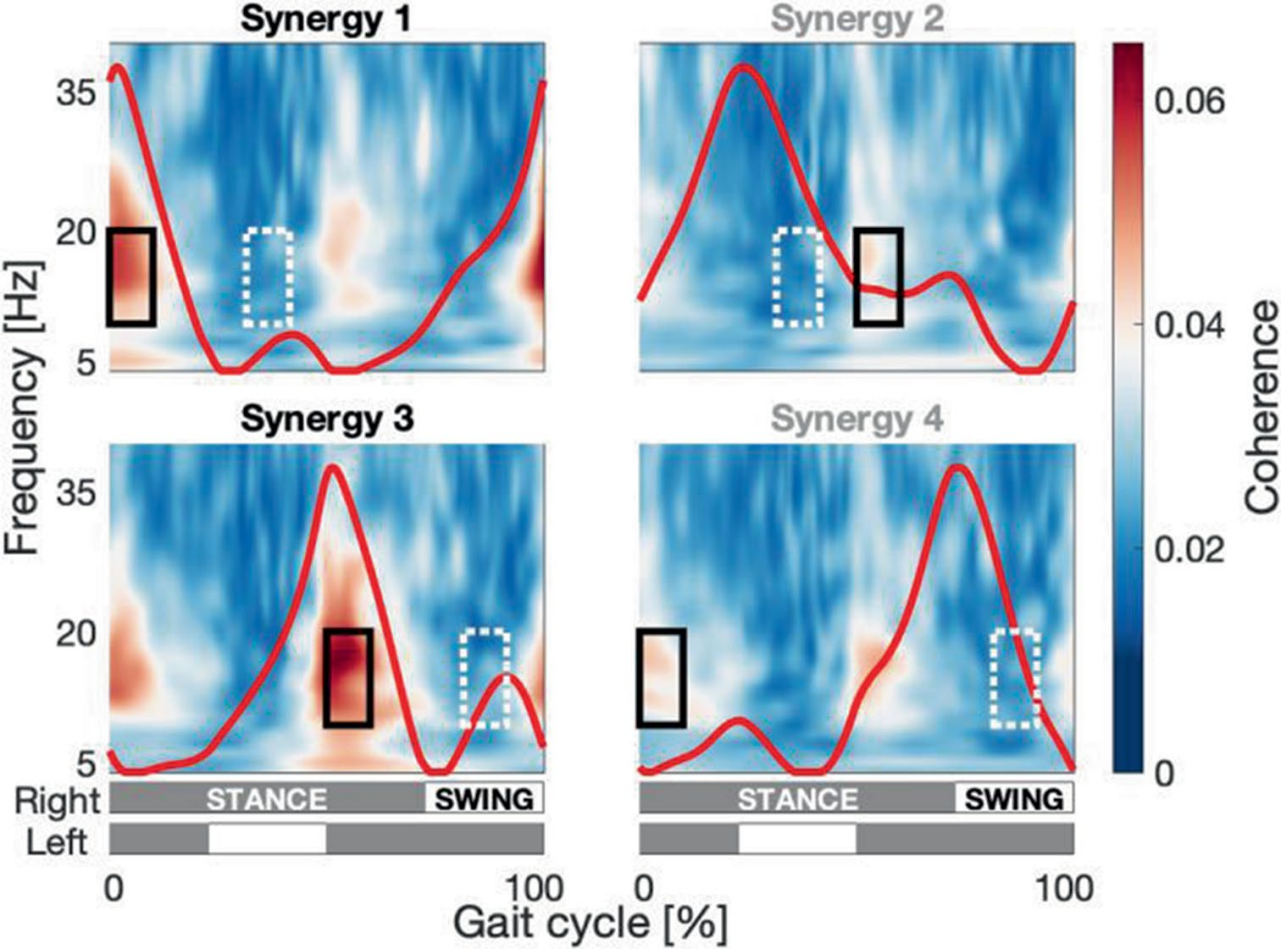
Sensor level cortico-synergy coherence over the gait cycle per synergy, pooled over conditions and averaged over the 7 central EEG channels (Cz, FC1, FC2, C3, C4, CP1, and CP2). The red line indicates the temporal activation patterns of the synergy analysis (Y in Eq. 1) pooled over conditions (Supplementary Material SM4). The boxes represent the windows of interest (black, solid) and contrast (white dotted) windows. The grey/white bars at the bottom indicate the stance (grey) and swing (white) phases of the left and right legs

Based on seven central channels overlaying the motor cortex (Cz, FC1, FC2, C3, C4, CP1, and CP2), cortico-synergy coherence was observed in the lower b-band frequency range (10–20 Hz), a frequency band commonly linked to corticospinal interaction and muscle coordination (Chung et al. 2017; Hussain et al. 2022; Kristeva et al. 2007; Reyes et al. 2017). As shown in Fig. 1, epochs with high coherence values were prominent during the initial part of the double-support phase, following the peak of the temporal synergy activation pattern, and lasted for approximately 10% of the gait cycle. By contrast, epochs of similar duration with minimal or no coherence were identified during the mid-single-support phase, following the peak of the temporal synergy activation pattern.

#### Source localisation

We first estimated a forward model (lead field) using an age-matched anatomical template derived from the Neurodevelopmental MRI Database (O’Reilly et al. 2021).This template was constructed using structural 3T MRIs scans of 75 18-month-old children. The MRI was segmented using finite element modelling yielding grey matter, white matter, cerebral spinal fluid, skull, and the scalp as probabilistic tissue maps. Their respective conductivities were set at 0.33, 0.14, 1.79, 0.01, and 0.43 S/m (Gabriel et al. 1996; O’Reilly et al. 2021). For every subject, using the cross-spectral density matrix of the EEG and muscle synergies averaged over the relevant and contrast epochs (10% initial double-support and 10% mid-single-support phases; Fig. 1), we estimated common spatial filters via the dynamic imaging of coherent sources (DICS) beamforming approach (Gross et al. 2001) in the lower b-band (10–20 Hz). Sources-of-interest were defined as locations at which the coherence during the relevant epoch differed significantly from that during the contrast epoch. To ensure that the detected sources were part of sensorimotor areas, we constrained them to the pre- and postcentral gyrus, according to the LPBA-40 atlas (Fillmore et al. 2015) containing bilateral primary sensory and motor cortices.

#### Source-level coherence analysis

The previous DICS spatial filters are optimal for the predefined epochs in the lower b-band. To obtain time series at the identified cortical sources covering the entire gait cycle and frequency spectrum, we employed a second reconstruction step. DICS spatial filters, optimised over the 5–40 Hz range for the entire gait cycle, were constructed for every significant voxel and averaged to form a single spatial filter for the entire source. This average spatial filter was transformed to only project to the dipole direction of maximal power using singular value decomposition. The resulting filter was used to construct the virtual source time series by projecting a 5–40 Hz bandpass filtered version of the pre-processed (not time normalised) EEG to source level activity.

Similar to the sensor level analysis, we computed the power-, cross-, and coherence time-frequency spectra for synergy and source time series and subsequently time normalised per stride. Here we would like to note that coherence is generally inflated at smaller sample sizes. In our data, the number of strides per BWS condition varied substantially (Table 1). To mitigate this bias, we ensured the trials were commensurate between the BWS conditions and age groups by applying a bootstrapping approach: The lowest number of strides $\:N$, recorded in any subject in any condition, was determined. Then, for every subject and condition 100 bootstrap samples, each containing $\:N$ strides, were drawn (without replacement). Coherence was estimated on each of them and subsequently averaged to get a coherence estimate per subject and condition.

### Statistics

#### Source localisation

The averaged cortico-synergy coherence of the start period of the double-support phases were compared to those of the mid-single-support phases using permutation testing based on the test statistic from a paired *t*-test (Maris et al. 2007; Maris and Oostenveld 2007). The two epochs were randomly partitioned for each subject and the test statistic for each source (voxel) was obtained. We repeated this for 2^15^ permutations to construct a Monte-Carlo distribution. The *p*-value for each voxel is based on the fraction of this null distribution that is at least as extreme as the test statistic from the original partition. We applied a False Discovery Rate (FDR) multiple comparison correction (Benjamini and Hochberg 1995) and set the significance threshold to a = 0.005.

#### Time-frequency coherence

The time-frequency cortico-synergy coherence values were averaged over the window of interest (i.e., coherence over the first 10% of gait cycle after foot contact in the 10–20 Hz frequency range). As the coherence tends to be beta distributed for very small sample size and small (true) coherence values, we applied a logit transform to stabilize variance. Testing for effects of load bearing and independent walking experience could then be realised via a mixed-design ANOVA. We set age (i.e., independent walking experience) as between-subjects factor and BWS as within-subjects factor. Post-hoc tests were performed to specify potential ANOVA interaction effects. For the latter we used non-parametric tests (Mann-Whitney U and Wilcoxon signed rank tests where appropriate) because normality could not be assured despite the logit transform. Main and interaction effects and post-hoc tests were assessed at an a-level of 0.05.

## Results

A total of 23 toddlers were recruited and 34 sessions were recorded. Gait was successfully recorded in 28 of these sessions (see *Supplementary Material SM2* for subject information). Only sessions with at least 15 strides in both BWS conditions were considered in the analysis. Additionally, one session was identified as an outlier due to its source-level coherence response to BWS, which was contrary to that of all other subjects in its group, and excluded to ensure the ANOVA could be appropriately applied. For results that include this subject, refer to *Supplementary Material SM7*. This left a total of 14 recording sessions over 12 subjects (FS: 7 subjects, 14.0 ± 1.9 months; FS + 6: 7 subjects, 19.3 ± 2.2 months; mean ± SD; see Table 1). Two of these subjects (P6 and P7) were recorded at both developmental stages, but these recordings were considered independent from one another as if they were separate subjects. Due to poor signal quality, 8% of all EMG recordings across subjects and BWS support conditions could not be reliably acquired and were therefore excluded from the analysis.

**Table 1:** Subject characteristics and number of strides

	FS group					FS + 6 group				
P#	Age (mo.)	WA (mo.)	BW (kg)	# strides		Age (mo.)	WA (mo.)	BW (kg)	# strides	
				Low BWS	High BWS				Low BWS	High BWS
P1	14.6	0.6	8.7	**27**	**23**					
P2	14.8	0.6	10.7	**53**	**57**					
P3	13.3	0.4	10.8	**35**	**45**					
P4	13.9	0.2	11.2	**225**	**15**					
P5	17.2	0.5	10.0	**267**	**25**					
P6	13.1	0.2	10.5	**161**	**40**	19.8	6.9	11.2	**286**	**53**
P7	10.9	0.3	10.3	**66**	**45**	16.5	5.9	11.3	**251**	**21**
P8						18.2	6.3	10.3	**245**	**34**
P9						20.8	5.5	13.0	**178**	**27**
P10						23.1	6.4	12.9	**196**	**21**
P11						19.4	4.5	11.0	**80**	**41**
P12						17.5	6.2	10.7	**167**	**20**

P# = subject number. WA = walking age (time since onset of independent walking). Mo. = Mo.ths. BW = body weight. # strides = numbers of strides. BWS = Body weight support

### Stride characteristics

While a total of about 4,200 strides were recorded, constraining our analysis to subjects with at least 15 strides in both BWS conditions reduced this to less than 3,000 strides in the analyses. Table 1 shows the distribution of these strides over the conditions and subjects. The 7 subjects in the FS group took on average 119 ± 97 strides in the low and 36 ± 15 in the high BWS condition. The 7 FS + 6 subjects took on average 200 ± 68 strides in the low and 31 ± 12 in the high BWS condition. It was significantly more challenging to get the toddlers to walk with a high level of BWS. Over all these strides, the right foot contact, left foot off, left foot contact, right foot off, and right foot contact gait events occurred on average at 0, 23, 50, 73, and 100% of the gait cycle, respectively (see *Supplementary Material SM3* for the distribution of these events over the gait cycle). These averages served as references to which the events in all strides were time normalised, enabling subsequent averaging over strides.

### Muscle synergies

**Fig. 2:**
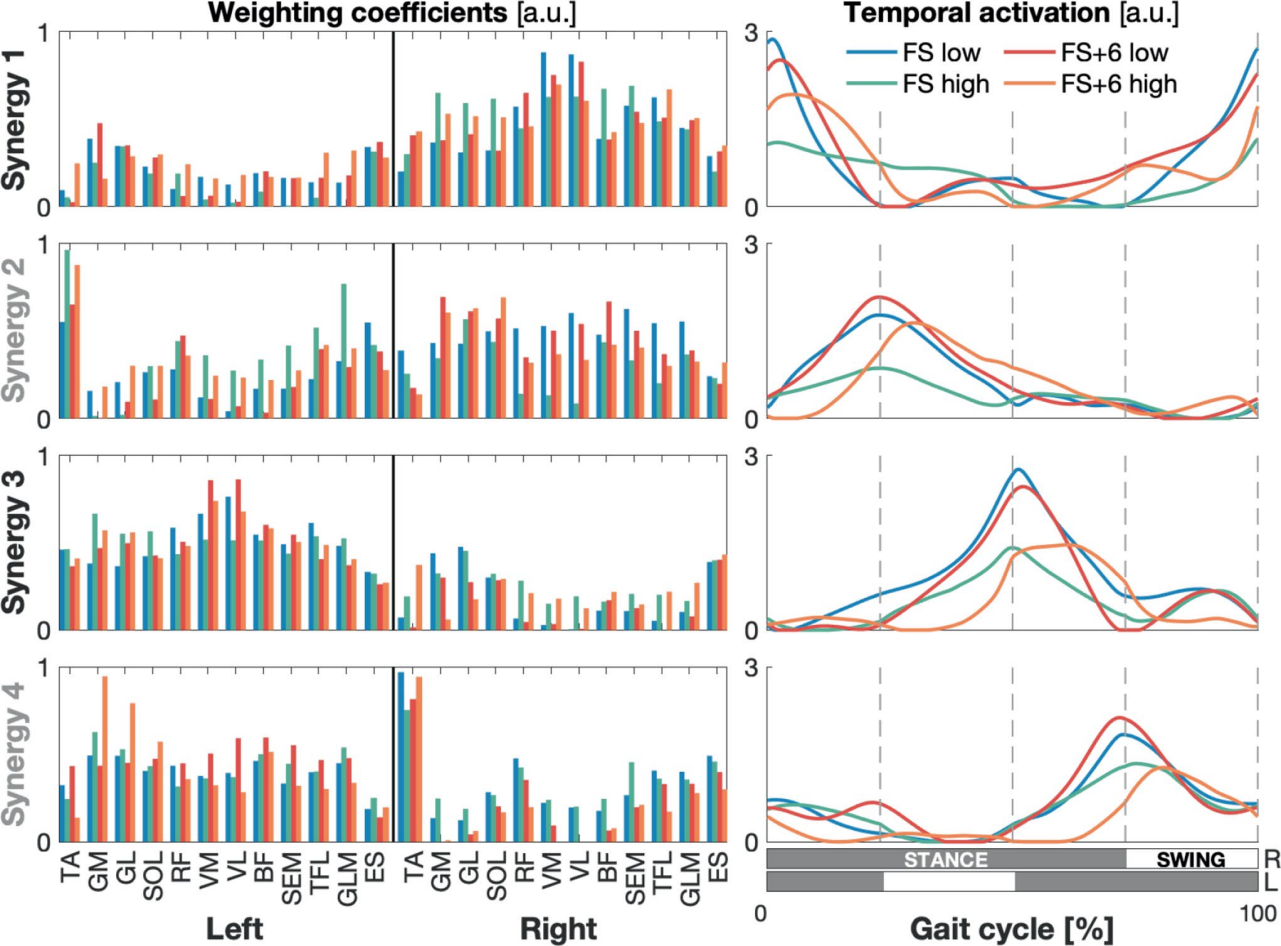
Muscle synergies per age group (FS and FS + 6 group) and BWS condition (low and high BWS). **Left column**: muscle weighting coefficients. **Right column**: temporal activation patterns. The grey/white bars underneath the temporal patterns indicate the stance (grey) and swing (white) phase of the right (top) and left (bottom) leg. **Rows**: The 4 synergies (S), where S1 & S3 represent the emerging and S2 & S4 the congenital synergies. TA, tibialis anterior; GL, gastrocnemius lateralis; GM, gastrocnemius medialis; SOL, soleus; RF, rectus femoris; VM, vastus medialis; VL, vastus lateralis; BF, biceps femoris; SEM, semitendinosus; TFL, tensor fasciae latae; GLM, gluteus maximus; ES, erector spinae. R, right; L, left.

In every condition the muscle activation patterns could be combined into four muscle synergies that reconstructed the EMG_e_ with 92–94% accuracy. The weighting coefficients did not differ substantially among the decompositions of the individual conditions (Fig. 2, left column; see also the *Supplementary Material SM4*). The temporal activation patterns were markedly lower in the high BWS conditions. Both weights and temporal activation patterns agreed with previous studies (Dominici et al. 2011; Kerkman et al. 2022). Synergies 2 & 4 (S2 & S4) bore comparison to the congenital synergies; synergies 1 & 3 (S1 & S3) matched the emerging synergies (Dominici et al. 2011; Zandvoort et al. 2022). Hence, we separated the temporal patterns of the synergies into two sets (Fig. 2, right column): one active around foot contact (S1 & S3), and the other one active around foot lift (S2 & S4). S1 and S2 primarily contained muscles of the right side of the body and S3 and S4 of the left side (Fig. 2, left column), except for the tibialis anterior in S2 and S4.

We would like to note that the here-obtained synergies were robust to different analysis approaches. Factorisation per condition (as used here) yielded qualitatively similar synergies to factorisation over the conditions pooled (see *Supplementary material SM4*). Additionally, the current method of factorising the averaged (over subjects) EMG gave comparable synergies to averaging the factorised EMG (not reported).

### Source localisation

**Fig. 3:**
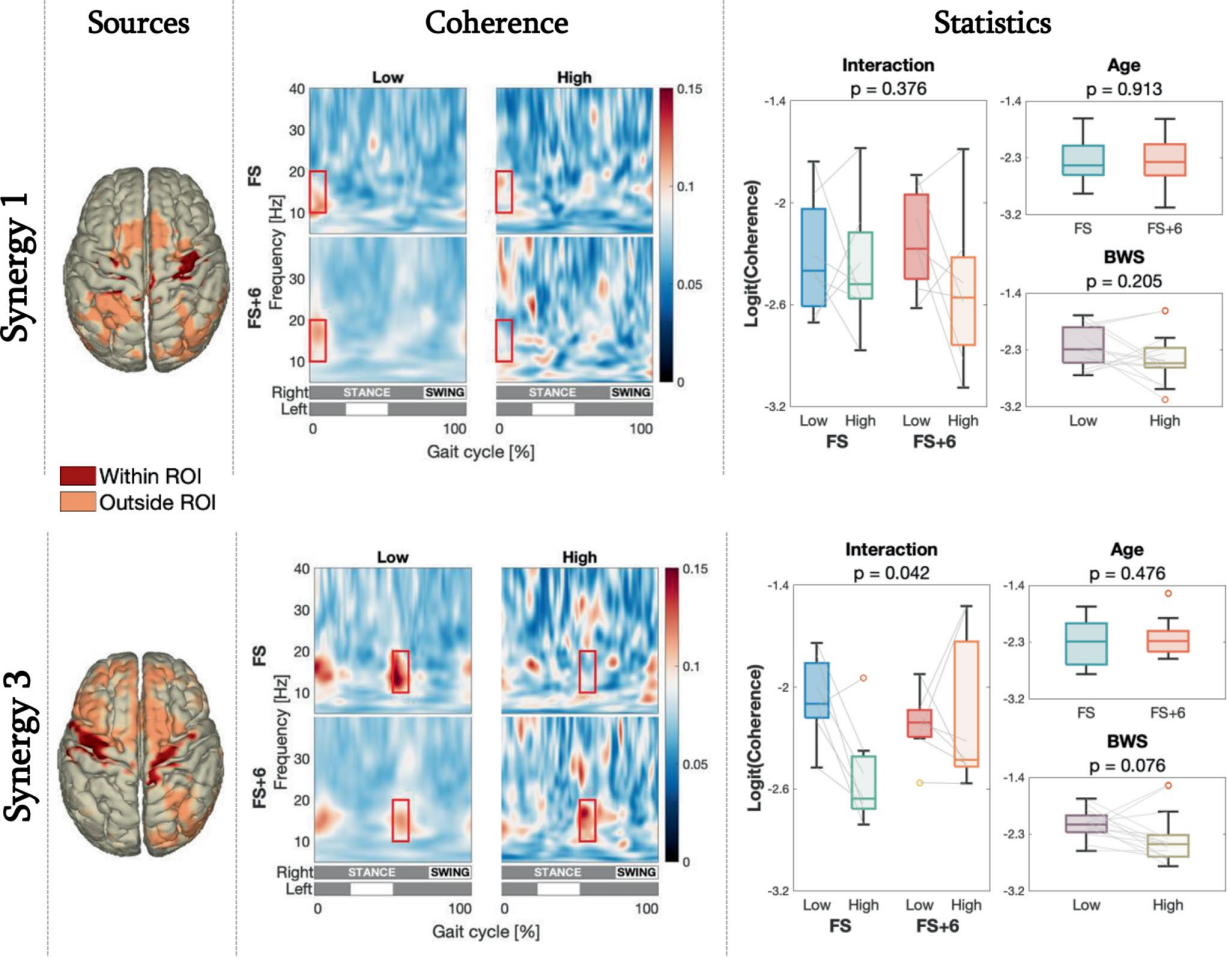
**Left column**: Localisation of cortical sources with significant coherence with the synergies 1 and 3. The coherence shown for each voxel is the average coherence pooled across conditions. (Dark red) Sources within the pre-defined region of interest (used in subsequent analyses). (Light red) Sources outside region of interest. Middle column: Time-frequency representations of the average cortico-synergy coherence over the gait cycle per condition. The grey/white bars at the bottom indicate the stance (grey) and swing (white) phases of the left and right legs. The red boxes indicate the windows of interest that were compared. **Right column**: Corresponding comparison of the logit-transformed coherence between conditions. The grey lines between low and high BWS conditions link the values of individual subjects. **Top row**: Synergy 1 observations. **Bottom row**: Synergy 3 observations

Sensor-level analysis over the pooled data indicated epochs with coherence in the low b-band after foot contact (at 0–10% of the gait cycle for S1 & S4 and at 50–60% for S2 & S3), and without coherence in the middle of the single support phase (at 32–42% of the gait cycle for S1 & S2, and at 82–92% for S3 & S4) (Fig. 1). Cortical sources exhibiting activity coherent with the synergies in the low b-band were only found for the emerging synergies S1 & S3 (*p* < 1.5·10^− 3^ and *p* < 1.8·10^− 3^ respectively, significant after FDR correction). These sources overlapped substantially with the a priori designated regions of interest, the pre- and postcentral gyri, where also the coherence was highest (Fig. 3, left column). We did not observe sources with significantly coherent activity to the congenital synergies S2 & S4.

### Cortico-synergy coherence

We focused on the cortical activation time series in the pre- and postcentral gyri for synergies S1 & S3. See *Supplementary Material SM5 & SM6* for the average relative power spectra of these sources and synergies. Figure 3 (middle column) depicts the corresponding time-frequency coherences, which were modulated over the gait cycle in the lower b-band. Notably, there was a focal surge following right and left foot contact for synergies S1 & S3, respectively. This pattern was present in the low BWS conditions for both synergies and also in the high BWS condition in the FS + 6 group for synergy S3.

A mixed-design ANOVA (Fig. 3, right column) on the window of interest indicated no main effect of age, BWS, nor an interaction effect on cortico-synergy coherence for synergy S1 (Age: *p* =.91, BWS: *p* =.21, interaction: *p* =.38). However, for synergy S3, the ANOVA showed a significant age × BWS interaction effect (*p* =.042). Post hoc tests indicated that higher BWS significantly decreased cortico-synergy coherence only in the FS group, where coherence was lower in high BWS condition compared to the other conditions (FS high vs.: FS low, *p* =.031; FS + 6 low, *p* =.038; FS + 6 high, *p* =.073). For the FS + 6 group, high BWS did not significantly affect coherence compared to the remaining conditions (FS + 6 high vs.: FS + 6 low, *p* =.938; FS low, *p* =.456), nor was the coherence significantly different during low BWS between the two age groups (FS low vs. FS + 6 low, *p* =.128).

## Discussion

The control of locomotion is governed by a combination of congenital and emerging muscle synergies, as demonstrated by the elicitable stepping responses of new-borns yet delayed onset of walking. The neural substrates that compose these synergies are (primarily) located in spinal centres termed CPGs (Hart and Giszter 2010; Lemay and Grill 2004; Pinto and Golubitsky 2006; Saltiel et al. 2001; Sun et al. 2022; Yang et al. 2019). Contrary to congenital synergies, the emerging ones also involve cortical contributions, manifested as cortico-synergy coherence (Zandvoort et al. 2022). When these synergies emerge, the onset of independent stepping is imminent, which is correlated to cortical maturation.

Here we targeted the activation of the sensorimotor network during gait by providing various levels of external body weight support. With less loading the decreased strain on the foot soles affects the cutaneous mechanoreceptors and the diminished stress on the (weight carrying) muscle-tendon complex affects the muscle spindle and Golgi tendon organ afferents, effectively reducing tactile and proprioceptive feedback. Our sensory intervention significantly decreased the cortico-synergy coherence in the initial developmental stage of independent walking, which was not the case in toddlers with 6 months of independent walking experience (Fig. 3, bottom row). This aligns with earlier observations that such an intervention yields changes in kinetic, kinematic, muscle activation, and control parameters in toddlers during the first independent steps (Dominici et al. 2007; Kerkman et al. 2022), most of which similarly diminished over gait development (Apte et al. 2018; Ivanenko et al. 2002, 2004). It hence appears that toddlers are only able to adequately adapt their gait control when a cortical resemblance of the emerging synergies is present. The cortical contribution seems to modulate the CPG patterns to achieve a properly balanced gait pattern. We propose that this modulation is the result of integration of sensory information.

### Modular control of locomotion

The reliance of gait on just a few basic activation patterns in the trunk and lower limbs has been demonstrated in various settings (Chvatal and Ting 2013; Dominici et al. 2011; Ivanenko et al. 2004, 2006; Zandvoort et al. 2021, 2022). In the current study we could identify these patterns regardless of independent walking experience and level of external support (Fig. 2). The four synergies resembled either the suggested congenital or the emerging pair based on their respective temporal and spatial patterns. The emerging synergies were mainly active directly after foot contact during the double support phases, when weight acceptance and transfer takes place. The temporal activation patterns were noticeably lower in the conditions with high BWS (Fig. 2, right column), which may be attributable to a reduced need for anti-gravity muscle activation. The activation patterns of the high BWS condition for the inexperienced walkers (the FS group) was less well defined and presented with less focal activation than that of the other conditions. Widening of activation patterns is also observed in cerebellar ataxia patients (Martino et al. 2015) and muscle spindle deficient mice (Santuz et al. 2019, 2023). Hence, it may reflect maladjustment to the imposed intervention due to a lack of sensorimotor integration.

### Function of the cortical contributions

There are several ways through which the cortex may contribute to gait control, of which we highlight three. (1) The cortex may (de-)activate the spinal CPGs, which then orchestrate the complex motor control. This could be a tonic signal, representing the voluntary intent to walk. (2) The cortex may leverage the CPGs’ structures and the cyclic pattern of gait. Gait is inherently unstable, requiring adaptability to sensory input to remain balanced. For this, the cortex might steer the CPGs’ ‘default’ motor patterns based on sensorimotor integration. Processing and planning of more abstract characteristics of the gait cycle (e.g., step width, swing/stance ratio, weight transfer speed) could then be handled by the cortex, while designated spinal centres compile the according motor commands. (3) Muscle activation could be a superposition of CPG and direct cortical efferents. In that case, however, the cortex will adjust individual muscles rather than sets of muscles.

We observed that the modular control of the inexperienced walkers (the FS group) with high BWS still resembled that of the other conditions (see green bars in Fig. 2, left column) and enacted a gait pattern, without having substantial cortical coherence with the emerging synergies (see FS high BWS plot in Fig. 3, middle bottom panel). This seems to suggest that also the emerging synergies are largely composed at the sub-cortical level. Furthermore, we observed the cortical coherence with S3 was affected by the sensory intervention. Moreover, the condition that presented without substantial cortical contribution has earlier been shown to display a maladaptive gait pattern, in contrast to the other conditions. Together this suggests the cortical contribution to the muscle output has a role in sensorimotor integration, effectively fine-tuning the CPG’s cyclic pattern to form more appropriate motor commands – supporting option (2) described above. The interpretation that the supraspinal drive steers the spinal structures is consistent with stimulation studies (Capelli et al. 2017), and with recent findings highlighting the importance of proprioceptive sensorimotor processing for the adaptive tuning of locomotion patterns (Santuz et al. 2022).

In fact, the role of cortical contribution in sensorimotor integration and its fine-tuning of the CPGs may also explain the observed modulation of coherence over the gait cycle. Coherence does not merely reflect synergy activation. It is pronounced only after foot contact (Fig. 3, middle column), whereas synergy activity already starts during the swing phase (Fig. 2, right column). Afferent information from the swing leg is strongly reduced by force on the contralateral stance leg through presynaptic inhibition (Hayes et al. 2012). Furthermore, during the swing phase the movement of the leg is endogenously prescribed, so its afferent information can be accurately anticipated by an internal model, reducing the need for complex sensorimotor processing. Once the foot contacts the ground, external forces act on the leg. This (sudden) sensory influx informs about the body’s current state but necessitates processing and integration into the motor plan for effective task execution. This idea is further aided by an increase in cortical excitability following cutaneous stimulation of the foot (Nielsen et al. 1997). Hence, the sudden onset of cortico-synergy coherence right after foot contact and its absence in the rest of the gait cycle would be expected if it represents sensorimotor integration.

### Development of emerging locomotor synergies

With the emergence of synergies S1 & S3, the motor repertoire for gait is ‘ready’ around the onset independent walking. This milestone moment requires a level of cortical maturation (Garwicz et al. 2009; Grillner 2011). The inexperienced walkers (the FS group) during the low BWS condition displayed all four synergies that also characterise older children and adult gait. Yet, our sensory intervention, in the form of body weight support, revealed a significant influence of sensory input in that group. It might be that sensorimotor integration has not been properly tuned causing an absence of cortico-synergy coherence when sensory input is reduced. This may potentially be aided by a still underdeveloped drive to the gamma motoneurons, which would cause an inability to refine muscle spindle sensitivity (Macefield 2021). While the intervention effect was observed only for S3, its absence in S1 may result from low statistical power (small sample size) due to the time sensitive nature and inherent challenges of doing measurements on toddlers.

Eventually the effect of the intervention disappears. After 6 months of walking experience (FS + 6) toddlers walking with high BWS displayed more well-defined synergies compared to the inexperienced walkers (FS). These also appeared to develop to be more robust to the sensory intervention. This is in line with observations that modular control of gait is robust to task conditions in adults (Chvatal and Ting 2013; Torres-Oviedo and Ting 2010). Increased robustness over time also adheres to the idea of the cortical drive initially shaping the CPG structure. In view of the Cheung et al. (2024) model, one can speculate that after an initial cortical drive to CPG centres, excitatory feedback loops from subsets of muscles to the CPG centres, and the concept of “neurons that fire together, wire together” eventually result in robust synergies.

## Conclusion

Already when taking their first independent steps a toddler’s sensorimotor cortex displays activity during gait that is coherent with newly emerging muscle synergies of the lower limb muscles. When toddlers with little independent walking practice experience a reduction in loading-related sensory information they are less able to adapt their gait pattern and the cortical contribution to the muscle output seemingly disappears. After six months of walking experience, the effect of gravitational loading on the cortico-synergy coherence largely disappears. We advocate the idea that coherent cortical activity tunes selected muscle synergies, potentially through modulation of dedicated spinal centres. This modulation is likely the result of sensorimotor integration that serves balancing gait.

## Electronic supplementary material

Below is the link to the electronic supplementary material.

Supplementary Material 1

## Data Availability

The data and code of this study can be made available upon request. Full availability may require a formal data sharing agreement, including a project outline and approval from the local ethics committee.
