# Supplementary material for: Exploring the cortical involvement in sensorimotor integration during early stages of independent walking
Source: Exp Brain Res. 2025 May 26;243(6):153. doi: 10.1007/s00221-025-07099-4 (PMC12106539; doi:10.1007/s00221-025-07099-4)
Supplement: Supplementary file 1 — Supplementary Material 1 [file 221_2025_7099_MOESM1_ESM.docx]

**Exploring the cortical involvement in sensorimotor integration during early stages of independent walking**

Ruud A. J. Koster, Coen S. Zandvoort, Jennifer N. Kerkman, Andreas Daffertshofer, Nadia Dominici

# Supplementary material

## Experimental Setup

| 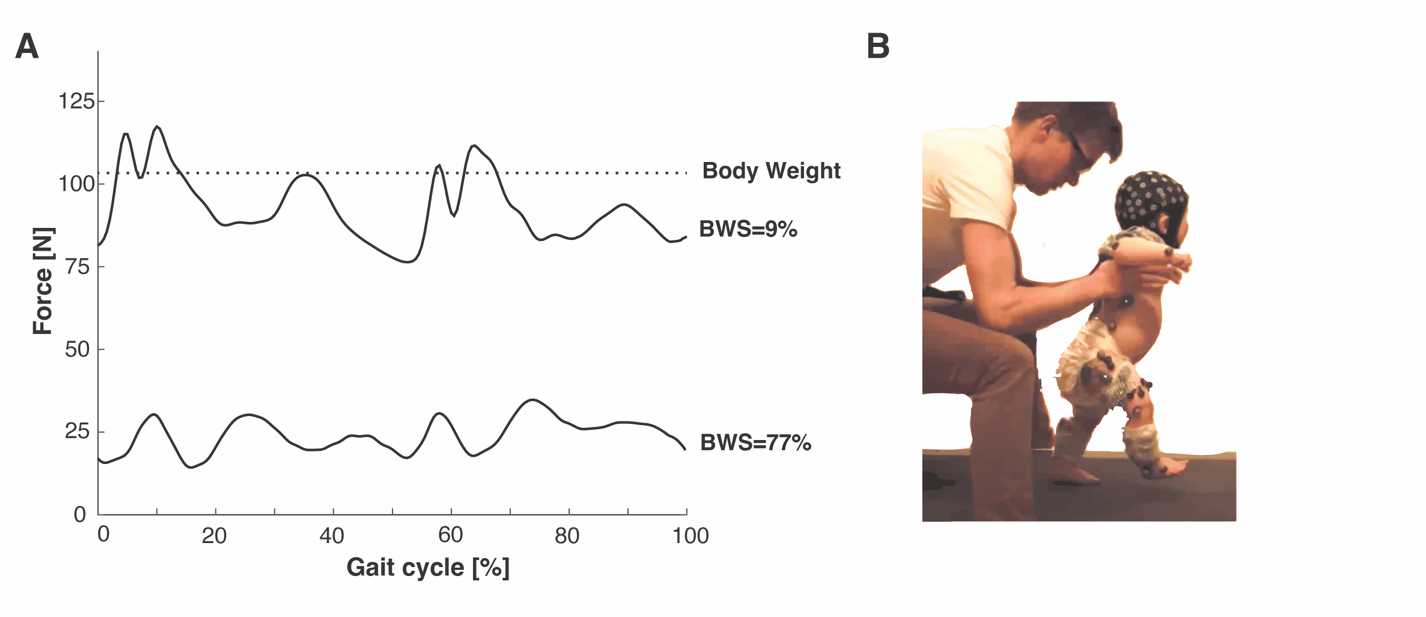 |
| --- |
| ***Figure SM1****: Experimental setup.* ***A)*** *Example of vertical ground reaction forces under the feet in a representative toddler at two levels of BWS. Dotted horizontal line indicate body weight. The amount of external body weight support was estimated as percent reduction of mean vertical force on the instrumented treadmill. BWS: Body Weight Support.* ***B)*** *The experimenter firmly supported the toddler’s trunk with both hands and applied an approximately constant upward force during several consecutive strides on the treadmill.* |

## Subject information

| Table SM2: Subject information of all subjects of whom strides were successfully recorded. P1-P11 were recorded at both stages of development. ‘Walking age’ = time since onset of independent walking in months. | | | | | | | | |
| --- | --- | --- | --- | --- | --- | --- | --- | --- |
| Characteristics of the recorded toddlers and the number of strides taken. | | | | | | | |  |
| Subject* | Gender | Age | Walking age | Body weight | Length | # Strides (mean %BWS) | |  |
|  |  | (mo.) | (mo.) | (kg) | (cm) | Low BWS | High BWS |  |
| FS group |  |  |  |  |  |  |  |  |
| P1 | F | 17.1 | 0.4 | 12.0 | 84 | 33 (26) | 2 (63) |  |
| P2 | F | 15.1 | 0.3 | 10.2 | 74 | 128 (14) | 11 (60) |  |
| P3 | F | 13.1 | 0.2 | 10.5 | 74 | 161 (19) | 40 (65) |  |
| P4 | F | 14.6 | 0.6 | 8.7 | 77 | 27 (15) | 23 (69) |  |
| P5 | M | 15.1 | 0.3 | 11.2 | 76 | 7 (27) | 85 (63) |  |
| P6 | M | 14.8 | 0.6 | 10.7 | 74 | 53 (23) | 57 (62) |  |
| P7 | M | 10.9 | 0.3 | 10.3 | 75 | 66 (22) | 45 (63) |  |
| P8 | F | 11.7 | 0.4 | 9.4 | 73 | 26 (13) | 22 (65) |  |
| P9 | F | 12.1 | 0.2 | 9.2 | 74 | 159 (12) | 71 (69) |  |
| P10 | M | 12.1 | 0.4 | 8.7 | 78 | 95 (1) | 2 (61) |  |
| P11 | M | 15.7 | 0.4 | 12.4 | 82 | 38 (1) | 0 (--) |  |
| P12 | F | 13.3 | 0.4 | 10.8 | 76 | 35 (15) | 45 (59) |  |
| P13 | F | 13.9 | 0.2 | 11.2 | 81 | 225 (20) | 15 (64) |  |
| P14 | M | 17.2 | 0.5 | 10.0 | 85 | 267 (25) | 25 (68) |  |
| FS+6 group |  |  |  |  |  |  |  |  |
| P1 | F | 23.1 | 6.4 | 12.9 | - | 196 (21) | 21 (60) |  |
| P2 | F | 19.4 | 4.5 | 11.0 | 84 | 80 (16) | 41 (66) |  |
| P3 | F | 19.8 | 6.9 | 11.2 | 82 | 286 (12) | 53 (67) |  |
| P4 | F | 20.1 | 6.2 | 10.3 | 81 | 145 (12) | 1 (58) |  |
| P5 | M | 20.3 | 5.6 | - | 79 | 44 (0) | 0 (--) |  |
| P6 | M | 23.6 | 9.4 | 12.2 | 88 | 25 (7) | 0 (--) |  |
| P7 | M | 16.5 | 5.9 | 11.3 | 79 | 251 (5) | 21 (69) |  |
| P8 | F | 17.5 | 6.2 | 10.7 | 82 | 167 (0) | 20 (63) |  |
| P9 | F | 18.2 | 6.3 | 10.3 | 83 | 245 (20) | 34 (64) |  |
| P10 | M | 17.4 | 5.7 | 10.5 | 80 | 25 (8) | 0 (--) |  |
| P11 | M | 20.8 | 5.5 | 13.0 | 96 | 178 (13) | 27 (60) |  |
| P15 | M | 18.6 | 5.7 | 12.4 | 81 | 0 (--) | 117 (78) |  |
| P16 | F | 19.3 | 6.2 | 12.5 | 81 | 293 (0) | 0 (--) |  |
| P17 | F | 19.7 | 6.7 | 10.4 | 84 | 161 (6) | 0 (--) |  |

** Subject numbers here do not coincide with the subject numbers in the main text.*

## Distribution of gait events

| 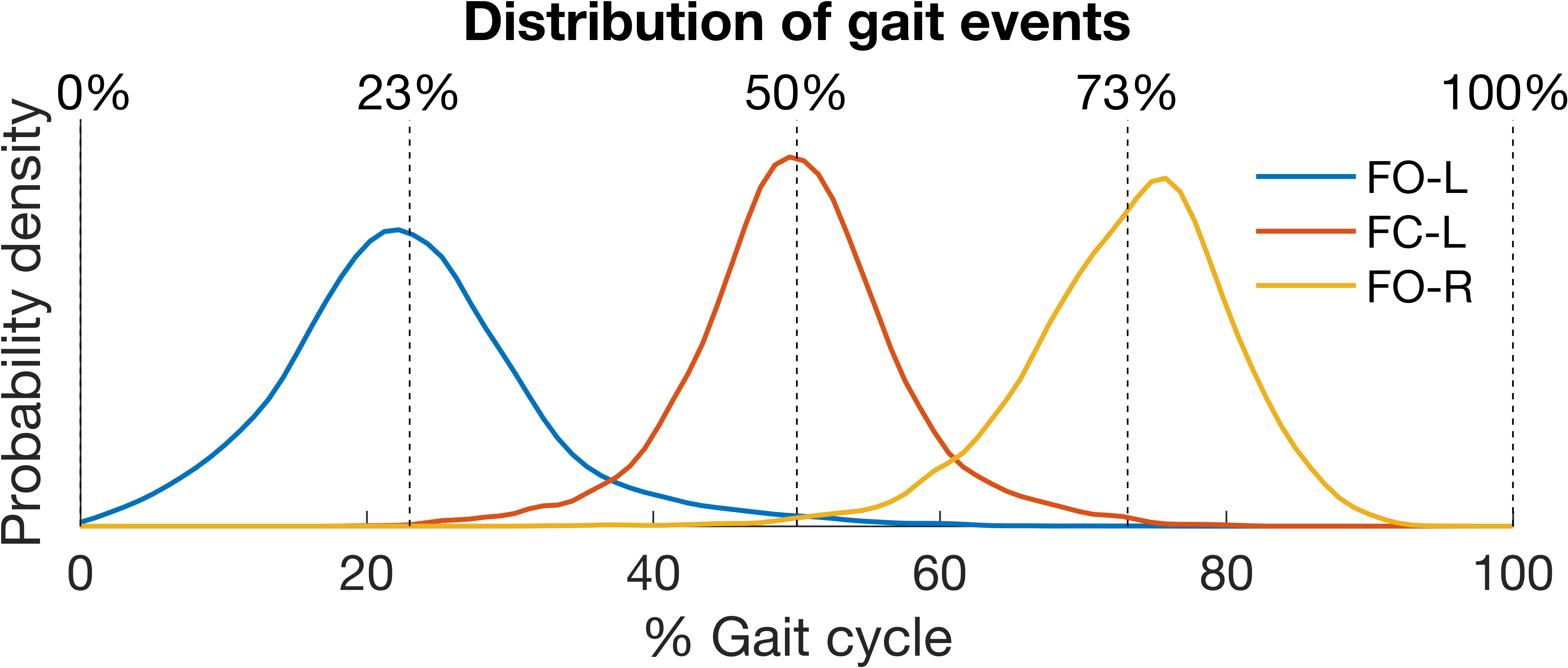 |
| --- |
| **Figure SM3**: Probability density estimates of the gait events over the gait cycle and their average occurrence over all strides. Strides were only considered if the events occurred in the right order. Probability densities of the right foot contact events are not included in the figure as they occurred exactly at 0 and 100% of the gait cycle for each stride by definition. Standard deviations of the other gait events were 8.9% (FO-L), 7.3% (FC-L), and 7.3% (FO-R). FC = foot contact; FO = foot off; -R = right; -L = left. The dashed lines and percentages at the top indicate the average occurrence of the events. Shown probability densities are kernel density estimators with a Gaussian kernel whose bandwidth was set according to Scott’s rule. |

## Comparison of synergies from pooled data vs data per condition

| 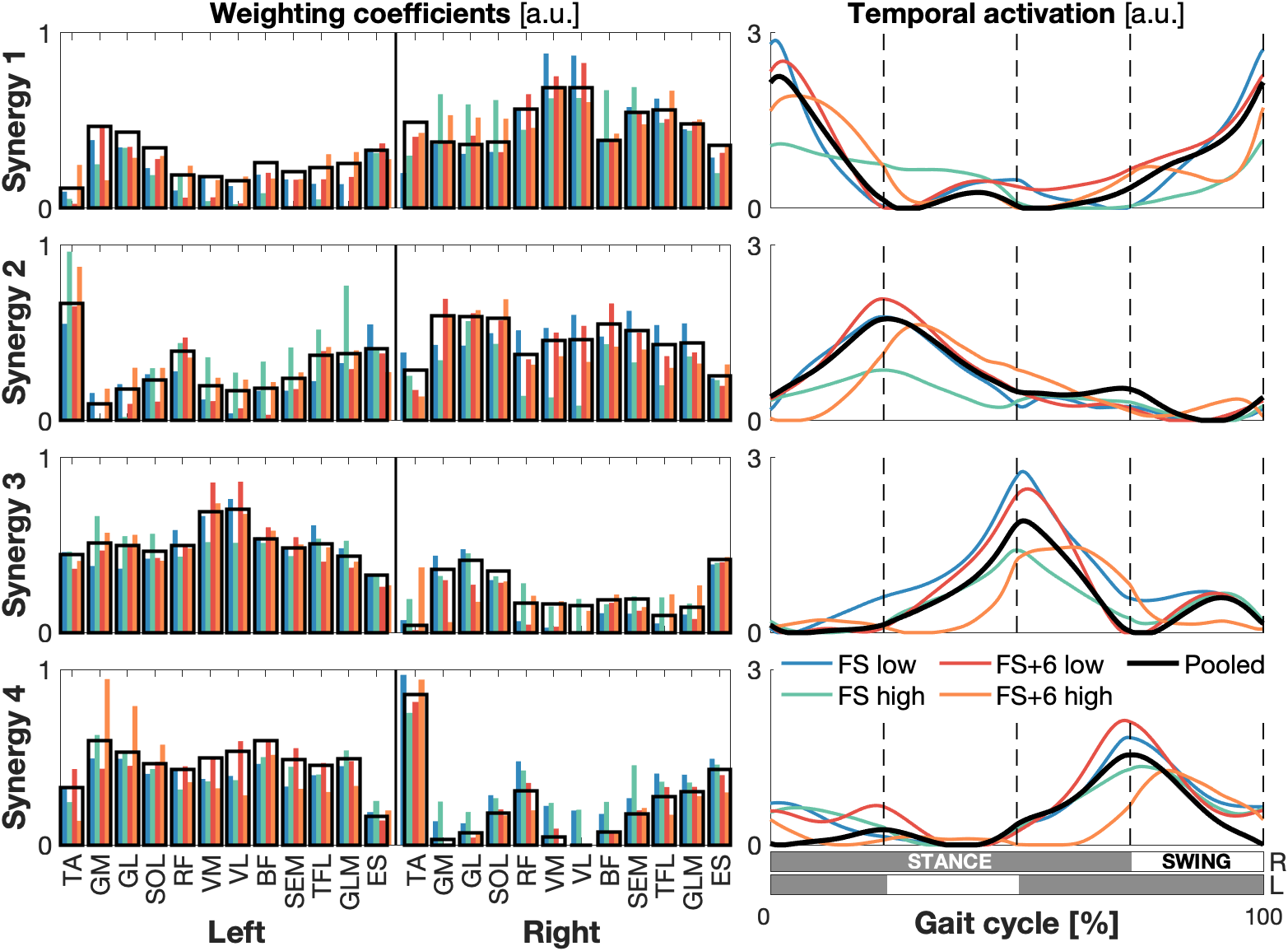 |
| --- |
| **Figure SM4**: Muscle synergies determined on the average stride activity (pooled over all conditions). Each row represents a single synergy. **Left column**: muscle weighting coefficients. **Right column**: temporal activation patterns. The grey/white bars underneath the temporal patterns indicate the stance (grey) and swing (white) phase of the right (top) and left (bottom) leg. The black bars (left) & lines (right) indicate the results obtained from the pooled data; the coloured bars (left) & lines (right) indicate the ‘per condition’ results as a reference (from Figure 2). Weighting coefficients and temporal activation patterns are by and large comparable between the pooled and un-pooled results. |

## Power spectra: Synergy 1 & corresponding cortical sources

| 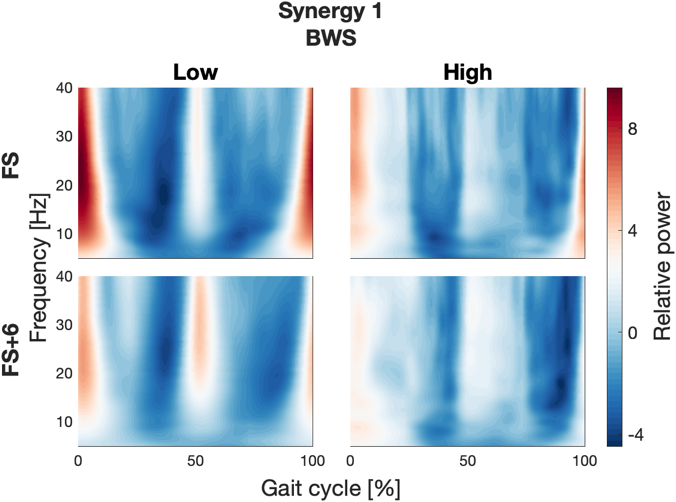 | 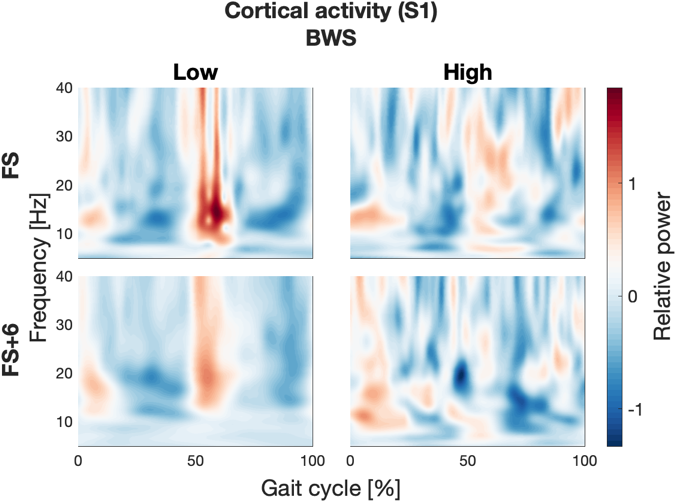 |
| --- | --- |
| **Figure SM5**: Relative power of the activity of synergy 1 (left) and the cortical sources identified to be coherent with synergy 1 (right), per condition. Power [dB] is expressed relative to the frequency’s mean. | |

## Power spectra: Synergy 3 & corresponding cortical sources

| 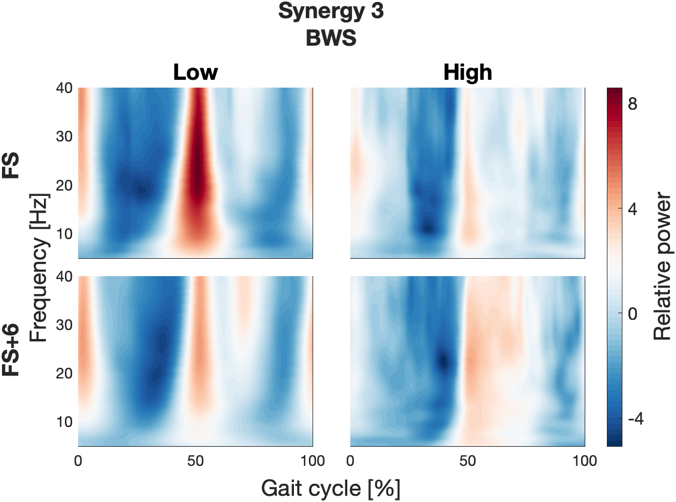 | 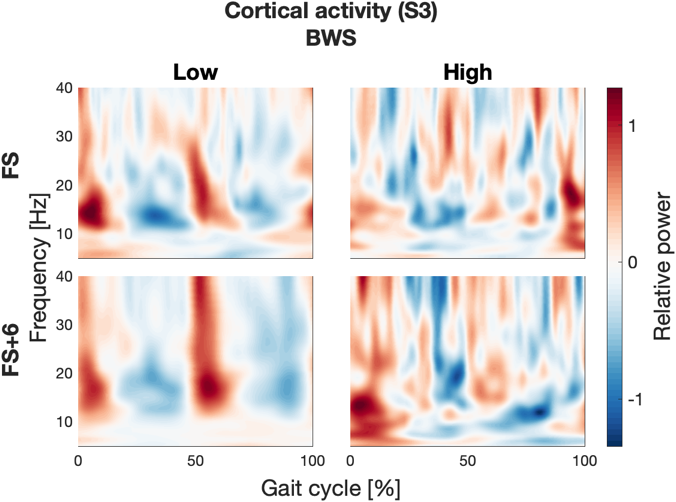 |
| --- | --- |
| **Figure SM6**: Relative power of the activity of synergy 3 (left) and the cortical sources identified to be coherent with synergy 3 (right), per condition. Power [dB] is expressed relative to the frequency’s mean. | |

## ANOVA results including outlier subject

The results without exclusion of the outlier subject are presented below. Note that coherence was obtained through bootstrapping to ensure commensurate sample size biases. As consequence of this random sampling the resulting values do not exactly coincide if this step is repeated. Hence, subject specific values in the boxplot here do not exactly match those in the boxplot in Figure 3 in the main text.

| **Synergy 1** | 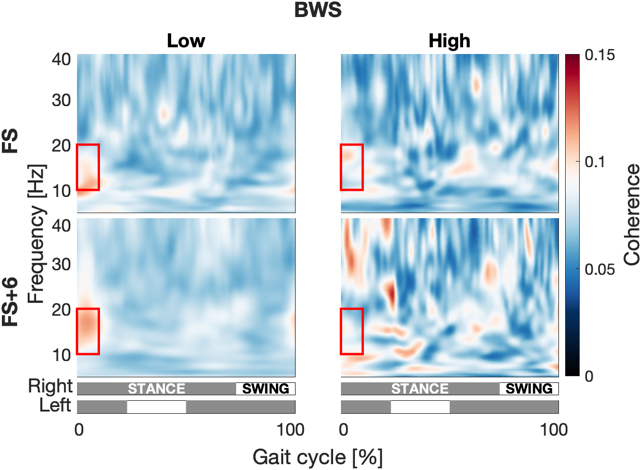 | 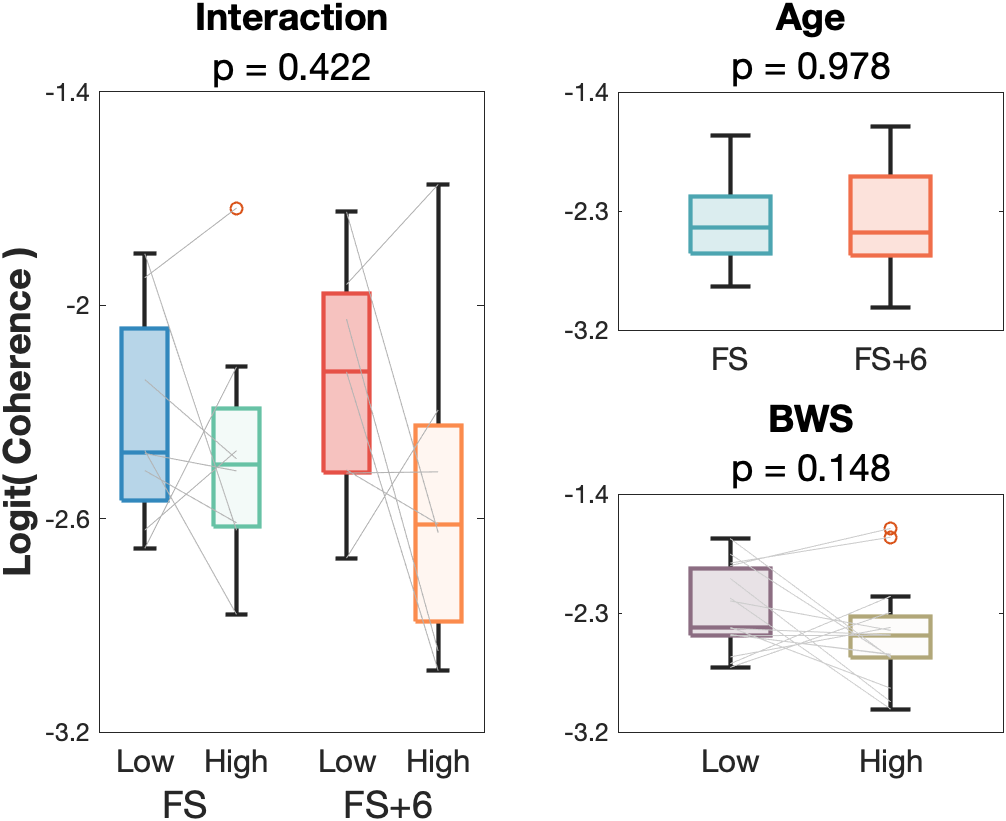 |
| --- | --- | --- |
| **Synergy 3** | 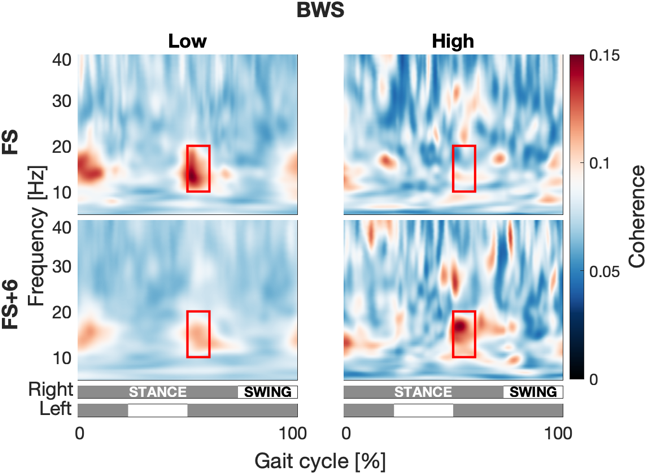 | 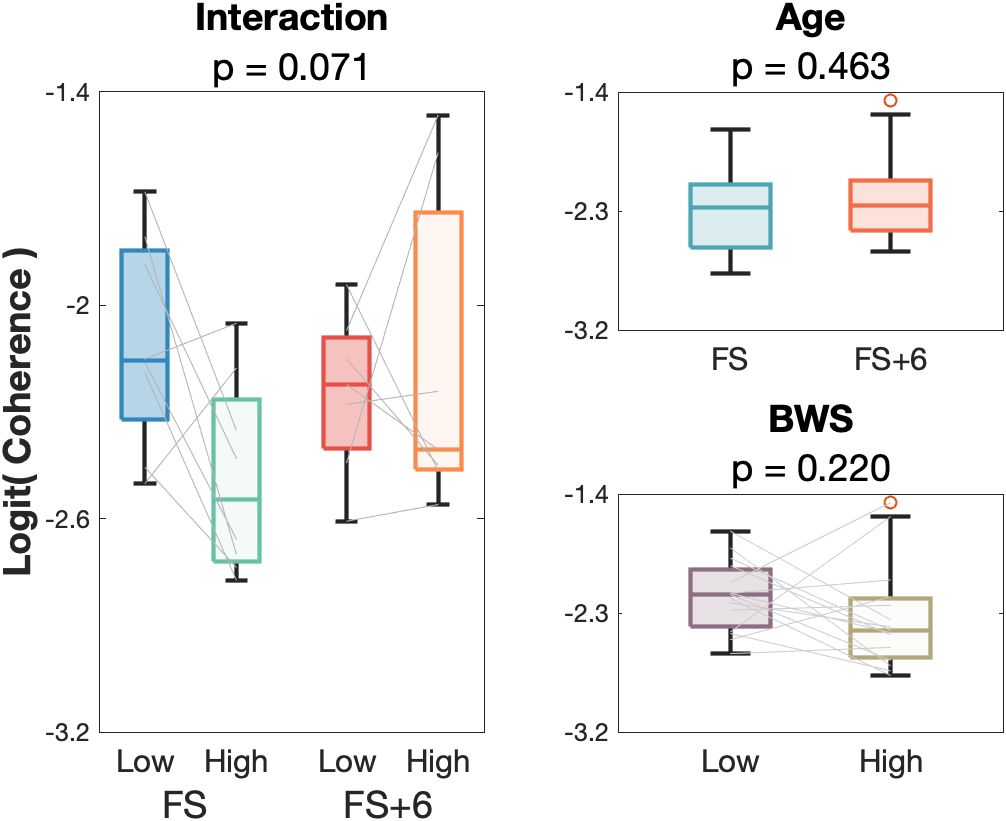 |
| **Figure SM7**: Results including outlier subject. **Left column:** Time-frequency representations of the average cortico-synergy coherence over the gait cycle per condition. The grey/white bars at the bottom indicate the stance (grey) and swing (white) phases of the left and right legs. **Right column:** Corresponding comparison of the logit-transformed coherence between conditions. The coherence presented is the mean coherence over the time-frequency window of interest (time: 50-60% of gait cycle; frequency: 10-20 Hz). The grey lines between low and high BWS conditions link the values of individual subjects. Results are based on all subjects with at least 15 strides in each condition. | | |
